# Optimized synthesis of novel prenyl ferulate performed by feruloyl esterases from *Myceliophthora thermophila* in microemulsions

**DOI:** 10.1007/s00253-017-8089-8

**Published:** 2017-01-11

**Authors:** Io Antonopoulou, Laura Leonov, Peter Jütten, Gabriella Cerullo, Vincenza Faraco, Adamantia Papadopoulou, Dimitris Kletsas, Marianna Ralli, Ulrika Rova, Paul Christakopoulos

**Affiliations:** 1 Division of Chemical Engineering, Department of Civil, Environmental and Natural Resources Engineering, Luleå University of Technology, 97187 Luleå, Sweden; 2 DuPont Industrial Biosciences, Nieuwe Kanaal 7-S, 6709 PA Wageningen, The Netherlands; 3 Taros Chemicals GmbH & Co. KG, Emil Figge Str 76a, 44227 Dortmund, Germany; 4 Department of Chemical Sciences, University of Naples “Federico II”, Via Cintia 4, 80126 Naples, Italy; 5 Institute of Biosciences and Applications NCSR “Demokritos,” Laboratory of Cell Proliferation and Aging, T. Patriarchou Grigoriou & Neapoleos, 15310 Athens, Greece; 6 Korres Natural Products, 57 Km National Road, 32011 Lamia, Athens Greece

**Keywords:** Feruloyl esterase, Transesterification, *Myceliophthora thermophila*, Prenyl ferulate, Antioxidant, DPPH, DCFH-DA

## Abstract

Five feruloyl esterases (FAEs; EC 3.1.1.73), FaeA1, FaeA2, FaeB1, and FaeB2 from *Myceliophthora thermophila* C1 and MtFae1a from *M. thermophila* ATCC 42464, were tested for their ability to catalyze the transesterification of vinyl ferulate (VFA) with prenol in detergentless microemulsions. Reaction conditions were optimized investigating parameters such as the medium composition, the substrate concentration, the enzyme load, the pH, the temperature, and agitation. FaeB2 offered the highest transesterification yield (71.5 ± 0.2%) after 24 h of incubation at 30 °C using 60 mM VFA, 1 M prenol, and 0.02 mg FAE/mL in a mixture comprising of 53.4:43.4:3.2 *v*/*v*/*v n*-hexane:*t*-butanol:100 mM MOPS-NaOH, pH 6.0. At these conditions, the competitive side hydrolysis of VFA was 4.7-fold minimized. The ability of prenyl ferulate (PFA) and its corresponding ferulic acid (FA) to scavenge 1,1-diphenyl-2-picrylhydrazyl (DPPH) radicals was significant and similar (IC_50_ 423.39 μM for PFA, 329.9 μM for FA). PFA was not cytotoxic at 0.8–100 μM (IC_50_ 220.23 μM) and reduced intracellular reactive oxygen species (ROS) in human skin fibroblasts at concentrations ranging between 4 and 20 μM as determined with the dichloro-dihydro-fluorescein diacetate (DCFH-DA) assay.

## Introduction

Ferulic acid (FA), along with other hydroxycinnamic acids (*p*-coumaric, caffeic, sinapic), has a widespread industrial potential due to its strong antioxidant activity. It is ubiquitous in nature as a component of plant cell walls offering linkage with lignin; in arabinoxylans, FA is esterified to the C-5 of α-L-arabinofuranose; in pectins, it is esterified to the C-2 of α1➔5-linked arabinofuranose or to the C-6 of β1➔4-linked galactopyranose, while in xyloglucans, it is found attached to the C-4 of α-D-xylopyranose (Kikugawa et al. 2012). FA is present in grains, fruits, and vegetables (Zhao and Moghadasian 2008). It has a broad spectrum of attractive biological properties including UV absorptive, antibacterial, antiviral, antiinflammatory, antithrombosis, and antitumor effects, while during the recent years, it is revealed that it may have beneficial effects against Alzheimer’s disease (Huang et al. 1988; Graf 1992; Kanski et al. 2002; Suzuki et al. 2002; Ou and Kwok 2004; Sultana et al. 2005; Vafiadi et al. 2008a; Barone et al. 2009). It is also suggested to suppress melanin generation by antagonizing tyrosine, which makes it a potential skin-whitening agent (Briganti et al. 2003; Chandel et al. 2011). Nevertheless, a major disadvantage of FA and other natural antioxidants is their poor solubility in both oil and aqueous media limiting their application in formulations intended for food, cosmetic, cosmeceutical, or pharmaceutical products.

A common way to alter solubility is by esterification or transesterification, with the latter requiring a prior activation of FA into an esterified derivative. Modification with sugars or glycerol results to more hydrophilic derivatives, whereas modification with fatty compounds results to more lipophilic products. Additionally to solubility, lipophilization has been shown to enhance the antioxidant activity of alkyl ferulate derivatives (Vafiadi et al. 2008b). Classic methods of esterification involve use of strong acids (concentrated sulfuric acid, hydrogen chloride) or expensive and toxic reagents as catalysts (boron trifluoride, aluminum chloride, trifluoroacetic anhydride, polyphosphate ester, neodymium oxide, dicyclohexylcarbodiimide, graphite bisulfate, etc.), high temperatures (150–250 °C), long reaction times, low yields, and tedious operations (Li et al. 2009). Process limitations include the heat sensitivity and oxidation susceptibility of FA; safety concerns for human health and the environment; and the high-energy consumption for purification, deodorization, and bleaching due to low selectivity (Kiran and Divakar 2001). The requirement for greener processes and the consumers’ preference for natural products demand the development of biotechnological sustainable and competitive processes for the production of interesting compounds with biological activities such as antioxidants. Enzymatic (trans)esterification is an attractive alternative as it offers mild conditions, use of greener solvents, and high selectivity.

During the past 15 years, the potential of feruloyl esterases (FAEs; EC 3.1.1.73) as biosynthetic tools has been underlined. FAEs represent a subclass of carboxylic acid esterases that are generally known to catalyze the hydrolysis of the ester bond between hydroxycinnamic acids and sugars as accessory plant cell wall-degrading enzymes. Based on their specificity towards monoferulates and diferulates, for substitutions on the phenolic ring and on their amino acid sequence identity, they have been classified into four types (A–D) (Crepin et al. 2004). The reported FAE-based modifications of hydroxycinnamic acids and their esters include their (trans)esterification with primary alcohols, e.g., 1-butanol, glycerol, or sugars in non-conventional media such as microemulsions of organic solvents characterized by low water content, solvent-free systems where the substrates function as reaction medium or single organic solvents. Specifically, lipophilization of FA has been performed by esterification with 1-pentanol using a type A FAE from *Aspergillus niger* in water-in-oil microemulsions (Giuliani et al. 2001). Transesterification of methyl ferulate (MFA) with 1-butanol has been reported in detergentless microemulsions of *n*-hexane:1-butanol:buffer using various FAEs such as StFae-A and StFae-C from *Sporotrichum thermophile* ATCC 34628, FoFae-I from *Fusarium oxysporum*, AnFaeA from *A. niger*, multienzymatic preparations such as Ultraflo L or Depol 740 L from *Humicola insolens*, and Depol 670 L from *Trichoderma reesei* resulting in varying yields (3–97%) (Topakas et al. 2003, 2004, 2005; Vafiadi et al. 2008a, b). The same reaction reached a conversion up to 90% when performed in a single solvent system (1-butanol:buffer) by immobilized Depol 740 L (Thörn et al. 2011). FAEs are generally less stable in non-conventional media and low water content than lipases, whereas they are more selective having higher substrate specificity (Zeuner et al. 2011). (Trans)esterification can be carried out by lipases only if the aromatic ring is not *para*-hydroxylated and the lateral chain is saturated. Thus, enzymatic (trans)esterification of hydroxycinnamoyl substrates can be obtained efficiently only by FAEs (Vafiadi et al. 2008a).

*Myceliophthora thermophila* (previously known as *S. thermophile*) is a thermophilic filamentous fungus that expresses a powerful consortium of enzymes able to break down lignocellulosic biomass (Karnaouri et al. 2014; Kolbusz et al. 2014). Its genome, which was entirely sequenced and annotated in 2011, encodes over 200 secreted carbohydrate-active enzymes (CAZy) and other enzymes of industrial interest (Berka et al. 2011). *M. thermophila* was developed into a mature protein production platform named C1. The main features of C1 include a low-viscosity morphology and high production levels (up to 100 g/L protein) in fed-batch fermentations providing thus an alternative to traditional fungal protein production hosts for cost-effective industrial applications (Visser et al. 2011). The genome of *M. thermophila* possesses six genes encoding enzymes belonging to the CE1 family of the CAZy database; four of which are FAEs (Hinz et al. 2009; Karnaouri et al. 2014). Three FAEs (FaeA1, FaeA2, and FaeB2) have been over-expressed in *M. thermophila* C1 and characterized (Kühnel et al. 2012). Sharing the same primary sequence with FaeB2, the type B FAE from *M. thermophila* ATCC 42464 (MtFae1a) has been heterologously expressed in *Pichia pastoris* and characterized (Topakas et al. 2012).

**Fig. 1:**
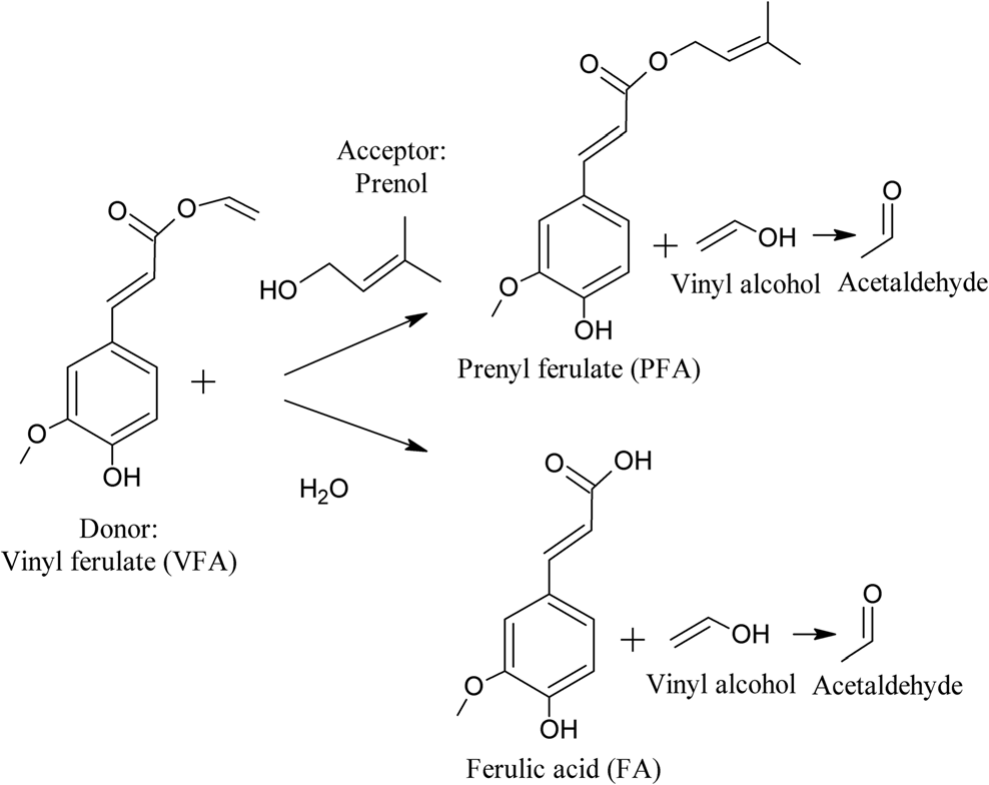
Scheme of **a** transesterification of VFA (donor) with prenol (acceptor). **b** Hydrolysis of VFA (natural reaction when water is present, observed as side reaction during transesterification)

In the present study, we synthesized a novel feruloylated derivative, namely, prenyl ferulate (PFA), by investigating different reaction parameters (medium composition, substrate concentration, enzyme concentration, pH, temperature, time, and agitation) in relation with the rate, the yield, and the product selectivity. Transesterification was performed using vinyl ferulate (VFA) as activated donor and prenol as acceptor, while a competitive side reaction of hydrolysis was observed with presence of water (Fig. 1). Prenol (3-methyl-2-buten-1-ol) is a natural occurring alcohol found in citrus fruits, berries, hops, tomato, grapes, passion fruit, and coffee, where it serves as a building block for terpenoids. In industry, it is used as a fragrance ingredient due to its fruity odor (Kabera et al. 2014). Polyprenols are abundant in wood and needles, such as pine and birch. They have attractive pharmacological effects that are based on their substitutive effect in the case of dolichol deficits, which are observed with chronic inflammatory, degenerative, and oncological diseases (Khidyrova and Shakhidoyatov 2002). The use of prenol as acceptor offers synthesis of a highly lipophilic derivative utilizing substrates of natural origin. In the same time, the use of novel FAEs derived from the thermophilic fungus *M. thermophila* offers an insight into their potential for efficient transesterification. These FAEs have been proved to be excellent hydrolytic enzymes; however, this is the first time that they are evaluated for their transesterification efficiency.

## Materials and methods

### Materials

VFA was prepared in 52% overall yield after three steps starting from FA. The procedure was modified from Mastihubova and Mastihuba (2013), offering >95% purity after column chromatography on silica gel based on ^1^H NMR and LC-MS. Methyl ferulate (MFA) was purchased from Alfa Aesar (Karlsruhe, Germany), while FA, prenol (99%), *n*-hexane (<0.02% water), *t*-butanol (anhydrous, ≥99.5%), MOPS solution 1 M, and other materials were purchased from Sigma-Aldrich (Saint Louis, USA).

### Enzymes

Feruloyl esterases FaeA1, FaeA2, FaeB1, and FaeB2 from *M. thermophila* C1 were over-expressed individually in low-background C1-expression hosts as reported previously (Kühnel et al. 2012; Visser et al. 2011). The C1-production strains were cultured aerobically in 2-L fermentors in a medium containing glucose, ammonium sulfate, and trace minerals. Protein production was done in a fed-batch system according to Verdoes et al. (2010). After fermentation, the enzyme-containing broth was separated from the biomass by centrifugation (15,000×*g* for 1 h at 4 °C) and filtration. The crude enzyme supernatant was concentrated eightfold, dialyzed against 10 mM potassium phosphate (pH 6.5), and was further lyophilized. MtFae1a from *M. thermophila* ATCC 42464 was recombinantly expressed in *P. pastoris* strain X33 as reported previously (Topakas et al. 2012). Samples of the culture broth were withdrawn after 3 days of incubation in 250-mL flasks at 20 °C; the biomass was removed by centrifugation (7000×*g* for 30 min at 4 °C); and the supernatant was 100-fold concentrated and dialyzed against 100 mM MOPS-NaOH, pH 6.0 using a tangential flow filtration system (10-kDa cutoff; PALL, NY, USA). Biochemical properties of the used FAEs are presented in Table 1.

**Table 1:** Biochemical characteristics of FAEs from *M. thermophila*

Enzyme	ID	Type	Subfamily	MW theoretical (kDa)	MW observed (kDa)	pI theoretical	pI experimental	Topt (°C)	pHopt	Source	Reference	FAE content (mg FAE/mg protein)	Specific activity (U/mg FAE)	
													MFA	PFA
FaeA1	JF826027.1^a^	A	SF5	29.2	29	5.9	~5.5	45	6.5	C1	Kühnel et al. (2012)	0.337	15.952 (0.108)	14.425 (0.045)
FaeA2	JF826028.1^a^	A	SF5	31.8	36	5.5	~5.2	40	7.5	C1		0.15	5.585 (0.091)	10.230 (0.009)
FaeB1	Sequence ID 71^b^	B	SF6	29.4	29	5.82	ND	ND	ND	C1	Gusakov et al. (2011)	0.5	8.557 (0.290)	9.149 (0.101)
FaeB2	JF826029.1^a^	B	SF6	31.4	33	4.6	~6.0	45	7.0	C1	Kühnel et al. (2012)	0.1	14.517 (0.917)	18.483 (0.939)
MtFae1a	AEO62008.1^a^	B	SF6	31.4	39	4.6	ND	50	7.0	ATCC 42464	Topakas et al. (2012)	0.422	10.491 (0.864)	13.907 (0.663)

Numbers in the parentheses are the estimates of standard errors

*ND* not determined

^a^GenBank ID

^b^Patent ID

### Enzyme and protein assays

Protein concentration was determined by the Pierce^™^ BCA Protein Assay (ThermoFisher Scientific, Waltham, USA). The FAE content (*w*/*w*) of the enzymatic preparations was determined by SDS-PAGE using a Novex Sharp pre-stained protein standard (ThermoFisher Scientific, Waltham, USA), followed by quantification using the JustTLC software (Sweday, Lund, Sweden). For the assessment of hydrolytic activity, a stock solution of substrate was prepared in dimethyl sulfoxide (DMSO). The activity was assayed using 1 mM MFA or PFA in 100 mM MOPS-NaOH, pH 6.0 and 0.005 mg FAE/mL enzyme load. Samples were incubated for 10 min at 45 °C without agitation. Reaction was ended by incubating the reaction mixtures at 100 °C for 5–10 min. All reactions were performed in duplicate and concomitant with appropriate blanks. One unit (1 U) is defined as the amount of enzyme (mg) releasing 1 μmol of FA per minute under the defined conditions. No substrate consumption was observed in the absence of esterase.

### Transesterification reactions

Transesterification reactions were performed in a ternary system of *n*-hexane:*t*-butanol:buffer forming detergentless microemulsions. Reactions were prepared by diluting adequate amount of donor in the mixture of *n*-hexane and *t*-butanol, followed by the addition of prenol and by vigorous shaking. Reaction was initiated by introducing the enzyme in the form of concentrated stock solution in buffer, followed by vigorous shaking until a stable one-phase solution was obtained. Transesterification was carried out in sealed glass vials at microscale (500 μL) in a temperature-controlled water bath. Medium composition, VFA concentration, prenol concentration, enzyme concentration, pH, and temperature were optimized. The effect of medium composition was studied at systems chosen according to previous reports (Topakas et al. 2005; Vafiadi et al. 2006b, 2008b) based on the phase diagram by Khmelnitsky et al. (1988). The effect of pH was studied using the following buffers at 100 mM concentration: sodium acetate (pH 4–6), MOPS-NaOH (pH 6–8), and Tris-HCl (pH 8–10). Optimal conditions obtained from each study were applied in subsequent experiments. Unless otherwise stated, reactions were performed at fixed conditions (50 mM VFA, 200 mM prenol, 0.02 mg FAE/mL FaeA1, FaeA2, 0.002 mg FAE/mL FaeB1, FaeB2, 0.04 mg FAE/mL MtFae1a, 40 °C, 100 mM MOPS-NaOH, pH 6.0, 8 h of incubation). Experiments that included agitation were performed in an Eppendorf thermomixer (Eppendorf, Hamburg, Germany). All reactions were performed in duplicate and concomitant with appropriate blanks. No donor consumption (<1%) was observed in the absence of esterase.

### Quantitative analysis

Analysis was performed by HPLC on a 100–5 C18 Nucleosil column (250 × 4.6 mm) (Macherey Nagel, Düren, Germany). Reaction mixtures were diluted with acetonitrile before analysis. Elution was done with 7:3 *v*/*v* acetonitrile:water for 10 min at a flow rate of 0.6 mL/min and room temperature. Absorbance was measured at 300 nm with a PerkinElmer Flexar UV/Vis detector (Waltham, USA). Retention times for FA, MFA, VFA, and PFA were 4.5, 6.1, 7.4, and 8.7 min, respectively. Calibration curves were prepared using standard solutions of feruloyl compounds in acetonitrile (0.1–2 mM). The sum of molar amounts of the donor and products at the end of reaction was always within a 5% error margin compared to the starting molar amount of the donor. The transesterification yield (or PFA yield) was calculated as the molar amounts of generated PFA compared to the initial amount of donor, expressed as a percentage. The overall yield was calculated as the molar amounts of PFA and FA compared to the initial amount of donor, expressed as percentage. Product selectivity was defined by the PFA/FA ratio (the molar concentration of produced PFA divided by the molar concentration of produced FA).

### Isolation of products and enzyme recovery

At the optimum conditions and after the reaction had been completed, the reaction mixture was diluted fivefold with buffer (100 mM MOPS-NaOH, pH 6.0), followed by an equal volume of *n*-hexane. After vigorous handshaking, two liquid layers were produced. The upper layer (organic, containing the lipophilic compounds) was separated and evaporated under vacuum, whereas the lower phase (aqueous, containing the hydrophilic compounds and the enzyme) was collected, buffer exchanged, and subsequently analyzed for activity. All obtained fractions were analyzed by HPLC.

### Structural characterization of prenyl ferulate

NMR spectroscopy was performed in DMSO-*d*_6_ with a Bruker Ascend Eon WB 400 spectrometer (Bruker BioSpin AG, Fällanden, Switzerland), ^1^H NMR (DMSO-*d*_6_, 400 MHz):δ 9.59 (s, 1H, ArO***H***), 7.53 (d, 1H, *J* = 16 Hz, −C***H***CHCOOR), 7.32 (d, 1H, *J* = 4 Hz, Ar***H***), 7.11 (dd, 1H, *J*_*1*_ = 2 Hz, *J*_*2*_ = 8.4 Hz, Ar***H***), 6.79 (d, 1H, *J* = 8.4 Hz, Ar***H***), 6.46 (d, 1H, *J* = 16 Hz, −CHC***H***COOR), 5.39–5.34 (m, 1H, −OCH_2_C***H***C<), 4.63 (d, 2H, *J* = 7.2 Hz, −O*C* ***H***_***2***_CHC<), 3.81 (s, 3H, −OC***H***_***3***_), and 1.72 (dd, 6H, *J*_*1*_ = 1.2 Hz, *J*_*2*_ = 14 Hz, −CHC(C***H***_***3***_)_2_).

### Antioxidant activity and cytotoxicity

The antioxidant activity of synthesized PFA as well as of its corresponding FA was monitored by the reduction in the optical density of 2,2-diphenyl-1-picrylhydrazyl (DPPH) radical. Serial dilutions of the test compounds were mixed with an equal volume of 1 mM DPPH in ethanol in U-bottomed 96-well plates and kept in the dark and at ambient temperature until measurement of absorbance at 520 nm at various time points. Human skin fibroblasts (HSFs; strain AG01523) were purchased from Coriell Institute for Medical Research (Camden, NJ, USA). Cells were cultured in monolayers and the possible cytotoxicity of PFA and FA was estimated by the 3-(4,5-dimethylthiazol-2-yl)-2,5-diphenyltetrazolium bromide (MTT) assay as previously described (Guldbrandsen et al. 2015). The antioxidant activity, expressed as the capacity to reduce the intracellular levels of reactive oxygen species (ROS), was assessed using the 2′,7′-dichlorofluorescein diacetate (DCFH-DA) assay. When HSFs were confluent, the medium was changed to serum-free Dulbecco’s modified Eagle medium (SF DMEM), and 18 h later, it was aspirated and renewed with phenol red and SF DMEM along with 10 μM of DCFH-DA. Following incubation with DCFH-DA for 1 h, serial dilutions of the test compounds were added and the fluorescence was measured in different time intervals for 480-nm excitation and 530-nm emission. The antioxidant activity of the compounds was visualized as reduction of DCF fluorescence and expressed as percent of control. Each experiment was conducted in triplicates.

## Results

### Effect of medium composition

**Fig. 2:**
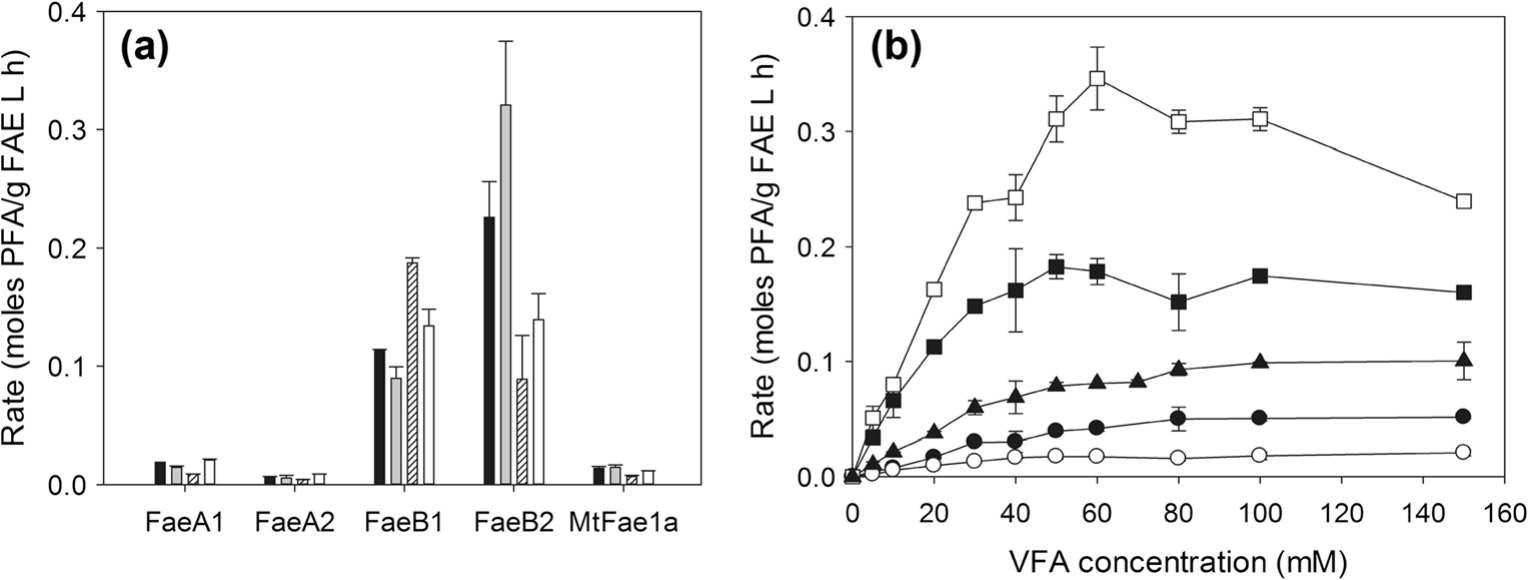
**a** Effect of medium composition on the rate. Reactions were performed in *n*-hexane:*t*-butanol:100 mM MOPS-NaOH using 50 mM VFA, 200 mM prenol at 40 °C, pH 6.0 for 8 h. *Black* system I (37.8:57.2:5.0 *v*/*v*/*v*), *gray* system II (53.4:43.4:3.2 *v*/*v*/*v*), *striped* system III (47.2:50.8:2.0 *v*/*v*/*v*), and *white* system IV (19.8:74.7:5.5 *v*/*v*/*v*). **b** Effect of VFA concentration on the rate. Reactions were performed at optimal medium composition for each enzyme, 200 mM prenol, 40 °C, pH 6.0 for 8 h. FaeA1 (*black circle*), FaeA2 (*white circle*), FaeB1 (*black square*), FaeB2 (*white square*), and MtFae1a (*black rectangle*) (rate is expressed as ×5 for FaeA1, FaeA2, and MtFae1a)

Transesterification was tested in four different compositions of the ternary system *n*-hexane:*t*-butanol:100 mM MOPS-NaOH, pH 6.0 monitoring the competitive hydrolysis of VFA as side reaction. Systems offering highest PFA concentration, rate, and yield were considered as optimal, system IV (19.8:74.7:5.5 *v*/*v*/*v*) for FaeA1 and FaeA2, system III (47.2:50.8:2.0 *v*/*v*/*v*) for FaeB1, and system II (53.4:43.4:3.2 *v*/*v*/*v*) for FaeB2 and MtFae1a (Fig. 2a). Generally, the highest PFA/FA molar ratio was achieved by FaeB2, while it was <1 for all tested FAEs and systems (0.095 for FaeA1, 0.034 for FaeA2, 0.301 for FaeB1, 0.513 for FaeB2, and 0.297 for MtFae1a at optimum conditions). Product selectivity was highest at lowest water content (system III, 2% water) for all tested FAEs; however, it was shown that such low water content was detrimental to the yield (except in the case of FaeB1). FaeB2 offered highest rate (0.321 mol PFA/g FAE L h), followed by FaeB1 (0.187 mol PFA/g FAE L h).

### Effect of VFA concentration

Under the optimal medium composition for each enzyme, the effect of VFA concentration on the transesterification was examined. Product selectivity was not affected by the variation in donor concentration (coefficient of variance ~2%), while higher concentrations resulted in higher rate but lower yield (Fig. 2b). The concentration of VFA that resulted in highest PFA concentration and rate was used as optimal donor concentration in subsequent experiments for each enzyme (80 mM for FaeA1, 50 mM for FaeA2 and FaeB1, 60 mM for FaeB2, and 100 mM for MtFae1a). Highest rate was 0.346 mol PFA/g FAE L h for FaeB2, followed by 0.183 mol PFA/g FAE L h for FaeB1 at optimal conditions.

### Effect of prenol concentration

**Fig. 3:**
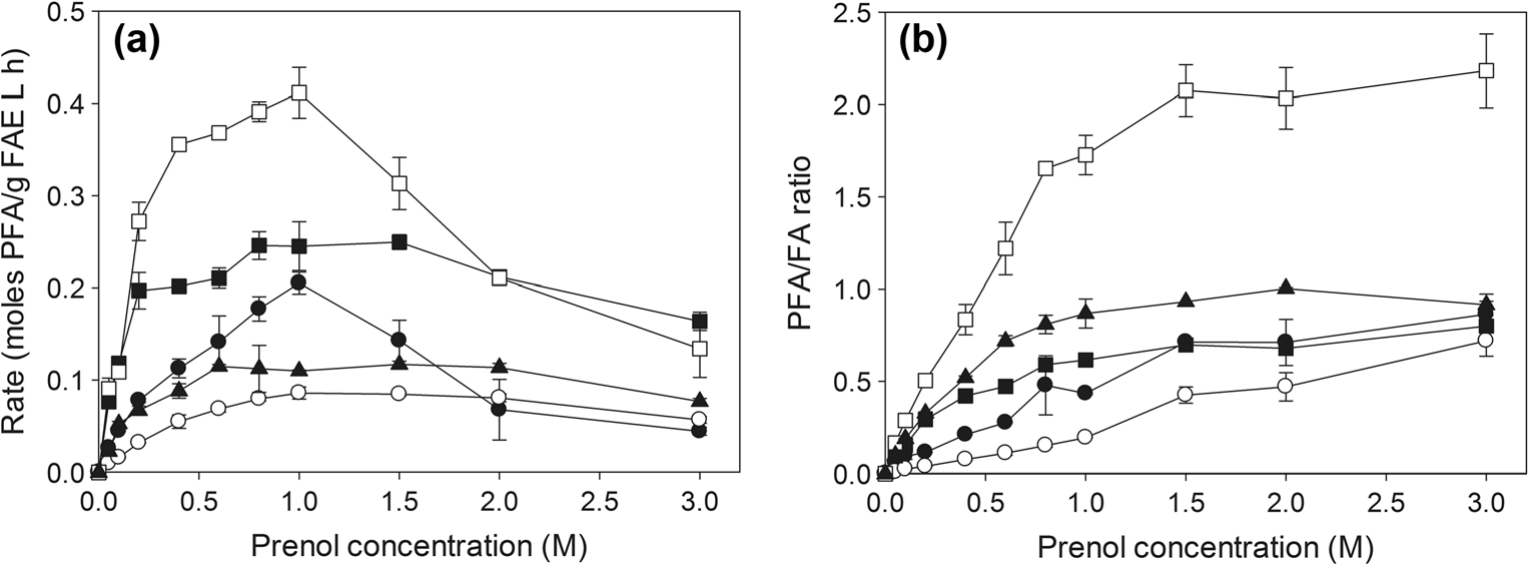
Effect of prenol concentration on the **a** rate and **b** product selectivity. Reactions were performed at optimal medium composition and VFA concentration for each enzyme, 40 °C, pH 6.0 for 8 h. FaeA1 (*black circle*), FaeA2 (*white circle*), FaeB1 (*black square*), FaeB2 (*white square*), and MtFae1a (*black rectangle*) (rate is expressed as ×5 for FaeA1, FaeA2, and MtFae1a in **a**)

The rate and yield increased remarkably by increasing the acceptor concentration. FaeB2 transesterified at highest rate (0.412 mol/g FAE L h) when 1 M prenol was used, followed by FaeB1 at 0.8 M prenol (0.246 mol/g FAE L h) (Fig. 3a). Optimal prenol concentration was implemented in subsequent experiments for each enzyme (1 M for FaeA1, FaeA2, and FaeB2 and 0.8 M for FaeB1 and 0.6 M for MtFae1a). Increase in prenol concentration remarkably increased transesterification against the side hydrolytic reaction, as more prenol molecules were available near the interface between the organic and water phase of the microemulsion, allowing more frequent transesterification instead of hydrolysis (Fig. 3b). Highest PFA/FA ratio (>1) was observed when FaeB2 was used, followed by MtFae1a. Nevertheless, higher product selectivity is generally observed at acceptor concentrations higher than 1.5 M, where rates are generally reduced. For instance, in the case of FaeB2, highest rate and yield were observed at 1 M prenol, but highest PFA/FA ratio was found at 1.5 M, where the overall yield decreased by 23.8%. On the contrary, MtFae1a seemed to have higher tolerance for prenol as the rate was practically constant between 0.5 and 2 M, although optimal performance was observed at only 0.6 M prenol.

### Enzyme kinetics

The apparent kinetic constants for each substrate were determined by fitting data regarding the effect of VFA and prenol concentration under standard reaction conditions (40 °C, pH 6.0) on the Michaelis-Menten equation using nonlinear regression (*p* < 0.0001) (Table 2). FaeB1 had highest affinity towards VFA and prenol (lowest *K*_*m*_), while FaeB2 had similar affinity with FaeB1 towards VFA and lower affinity towards prenol comparing to FaeB1 and MtFae1a. However, FaeB2 catalyzed fastest the transesterification (highest *v*_max_) and was most efficient catalyst for both substrates (highest *K*_*m*_/*k*_cat_). MtFae1a had lower affinity for VFA and was approximately 300 times less efficient catalyst comparing to FaeB2. Type A FAEs had the lowest affinity towards prenol, showing low efficiency in the synthesis of PFA (Fig. 3).

**Table 2:** Apparent kinetic constants

Enzyme	VFA				Prenol			
	*v*_max_ (mol/g FAE L h)	*K*_*m*_ (mM)	*k*_cat_ (min^−1^ g FAE^−1^)	*k*_cat_/*K*_*m*_ (M^−1^ min^−1^ g FAE^−1^)	*v*_max_ (mol/g FAE L h)	*K*_*m*_ (mM)	*k*_cat_ (min^−1^ g FAE^−1^)	*k*_cat_/*K*_*m*_ (M^−1^ min^−1^ g FAE^−1^)
FaeA1	0.015 (0.002)	54.5 (11.7)	0.3698	6.783	0.073 (0.008)	831.4 (173.0)	1.752	2.107
FaeA2	0.0052 (0.001)	46.5 (18.1)	0.156	3.358	0.025 (0.002)	504.3 (92.8)	0.735	1.457
FaeB1	0.284 (0.020)	30.6 (4.8)	68.6	2245.1	0.270 (0.010)	113.2 (18.6)	65.2	492.2
FaeB2	0.455 (0.054)	31.6 (9.6)	125.2	3956.7	0.516 (0.035)	228.6 (53.1)	141.7	619.9
MtFae1a	0.033 (0.005)	81.2 (22.3)	0.488	6.604	0.027 (0.001)	186.9 (31.4)	0.440	2.356

Numbers in the parentheses are the estimates of standard errors

### Effect of enzyme concentration

**Fig. 4:**
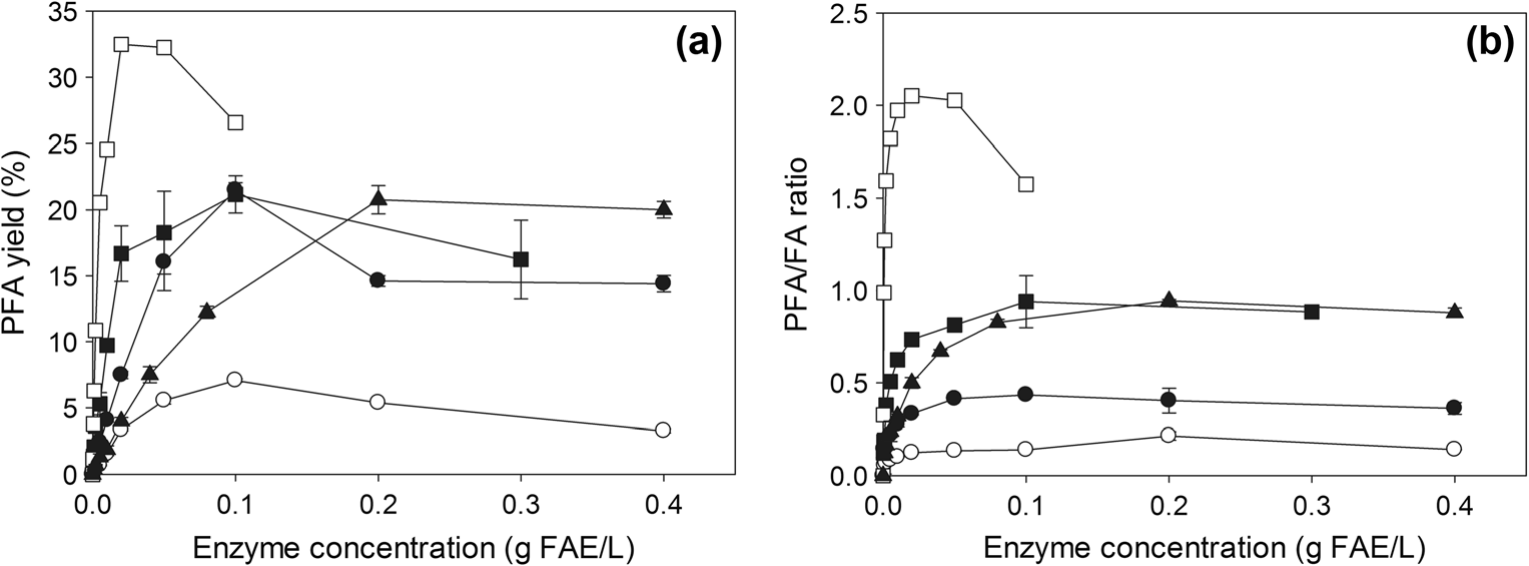
Effect of enzyme concentration on the **a** yield and **b** product selectivity. Reactions were performed at the optimum medium composition and substrate concentration for each enzyme, 40 °C, pH 6.0 for 8 h. FaeA1 (*black circle*), FaeA2 (*white circle*), FaeB1 (*black square*), FaeB2 (*white square*), and MtFae1a (*black rectangle*)

In most industrial applications, cost of enzyme production and operational costs are usually the limiting factors. Improvement on the yield and reduction of reaction time occur when the enzyme concentration increases, leading to a reduction of operation costs; however, the process cost in terms of enzyme production increases significantly, too. With the addition of more enzyme, the rate (mol/g FAE L h) decreased exponentially (data not shown), but the PFA yield reached a peak at different enzyme concentration for each tested FAE (Fig. 4a). FaeB2 was able to synthesize PFA at highest yield (32.5%) and rate (0.122 mol/g FAE L h) at minimal enzyme load of 0.02 mg FAE/mL. FaeA1, FaeB1, and MtFae1a achieved similar PFA yields (~21%) at optimal FAE concentration of 0.1 mg/mL for FaeA1 and FaeB1 and 0.2 mg/mL for MtFae1a. Regarding product selectivity, only FaeB2 offered a ratio >1, while MtFae1a and FaeB1 achieved a value of 0.938 at optimal conditions (Fig. 4b). In low enzyme concentrations, an increase substantially affected the PFA/FA ratio, as more enzyme molecules were available near the interface of the organic and aqueous phase being able to catalyze the transesterification. However, for most FAEs, the yield and product selectivity stabilized or decreased over 0.1–0.2 mg FAE/mL. Optimal conditions were implemented in subsequent experiments.

### Effect of pH

Although the water content in the reaction is low, the pH of the aqueous solution may influence the ionization state of the residues of the enzymes’ active site. Rate, yield, and product selectivity were observed in transesterification reactions performed at pH range of 4–10. Environment that offered highest PFA concentration, yield, and rate was considered as optimal and proved to be 100 mM MOPS-NaOH buffer at pH 6.0 for FaeB2 and FaeA2, pH 7.0 for FaeB1, and pH 8.0 for FaeA1 and MtFae1a. In these conditions, the PFA/FA ratio was equal to 0.692 for FaeA1, 0.295 for FaeA2, 1.119 for FaeB1, 2.298 for FaeB2, and 1.670 for MtFae1a. Product selectivity was affected by pH, as highest PFA/FA ratio was observed at pH 4.0 for FaeA2, pH 5.0 for FaeB1, pH 6.0 for FaeB2, and pH 8.0 for FaeA1 and MtFae1a. However, in the cases of FaeA2 and FaeB1, low pH was detrimental to the yield.

### Effect of temperature

**Fig. 5:**
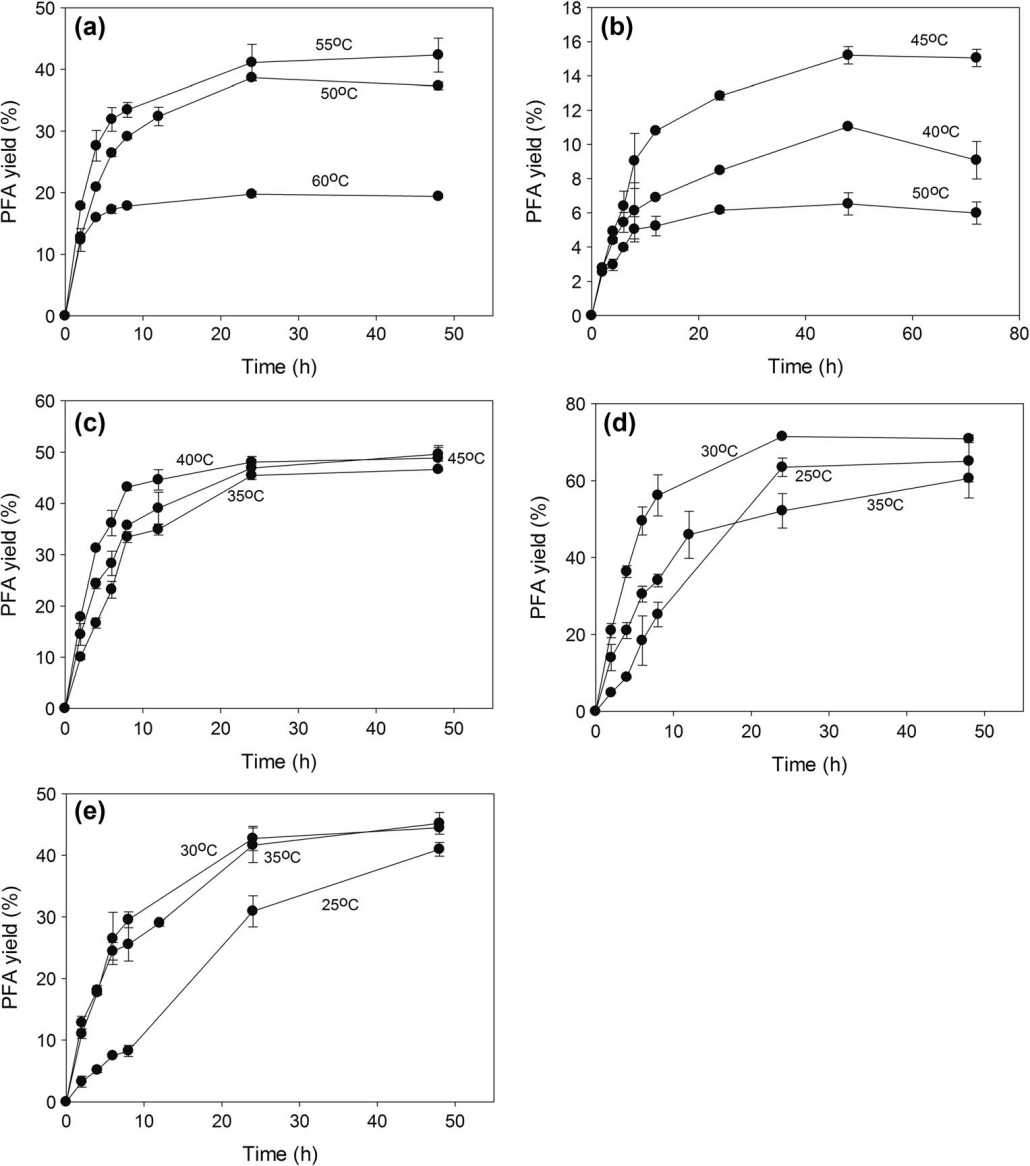
Effect of temperature on the yield. Reactions were performed at optimal medium composition, substrate concentration, enzyme concentration, and pH for each enzyme. **a** FaeA1, **b** FaeA2, **c** FaeB1, **d** FaeB2, and **e** MtFae1a

Transesterification was monitored at different temperatures (25–60 °C) with respect to time (Fig. 5). Among all, FaeB2 had the highest PFA yield (71.5%), rate (0.211 mol PFA/g FAE L h), and product selectivity (2.373) at 30 °C after 24 h of incubation, achieving a 100% conversion of VFA to products. FaeA1 and FaeA2 appeared to be more thermophilic with optimal performance at 55 and 45 °C, respectively. Interestingly, although both FaeB2 and MtFae1a performed optimally at 30 °C, FaeB2 appeared to inactivate at 35 °C, while MtFae1a had the same profile as at 30 °C during transesterification. At optimal temperature, FaeA1, FaeB1, and MtFae1a reached a PFA yield of 40–48% out of 93.9, 83.1, and 63.2% overall yield after 24 h, respectively. Lowest yield was demonstrated by FaeA2 15.2% after 48 h out of 82.2% overall yield. A summary on the obtained parameters for each enzyme is presented in Table 3.

**Table 3:** Summary of optimal conditions and obtained parameters

Enzyme	FaeA1	FaeA2	FaeB1	FaeB2	MtFae1a
Optimized conditions^a^					
Water content (%)	5.5	5.5	2.0	3.2	3.2
VFA concentration (mM)	80	50	50	60	100
Prenol concentration (M)	1	1	0.8	1	0.6
Enzyme concentration (g FAE/L)	0.1	0.1	0.1	0.02	0.2
pH	8	6	7	6	8
Temperature (°C)	55	45	40	30	30
Time (h)	24	48	24	24	24
Obtained parameters					
PFA concentration (mM)	32.9 (2.4)	7.6 (0.3)	24.1 (0.2)	42.9 (0.1)	42.7 (2.0)
PFA yield (%)	41.1 (3.0)	15.2 (0.5)	48.1 (0.4)	71.5 (0.2)	42.7 (2.0)
Overall yield (%)	93.9 (5.4)	82.2 (0.5)	83.1 (3.0)	102.0 (4.7)	63.2 (1.6)
Rate (mol PFA/g FAE L h)	0.014 (0.001)	0.0016 (0.0001)	0.010 (0.000)	0.089 (0.000)	0.0089 (0.0004)
Initial rate (mol PFA/g FAE L h)	0.053 (0.003)	0.0057 (0.001)	0.030 (0.002)	0.182 (0.008)	0.022 (0.004)
PFA/FA ratio	0.778 (0.021)	0.227 (0.011)	1.378 (0.093)	2.373 (0.362)	1.700 (0.041)

Numbers in the parentheses are the estimates of standard errors

^a^Concentrations are expressed as in total volume of reaction (500 μL)

### Effect of other donors and agitation

**Fig. 6:**
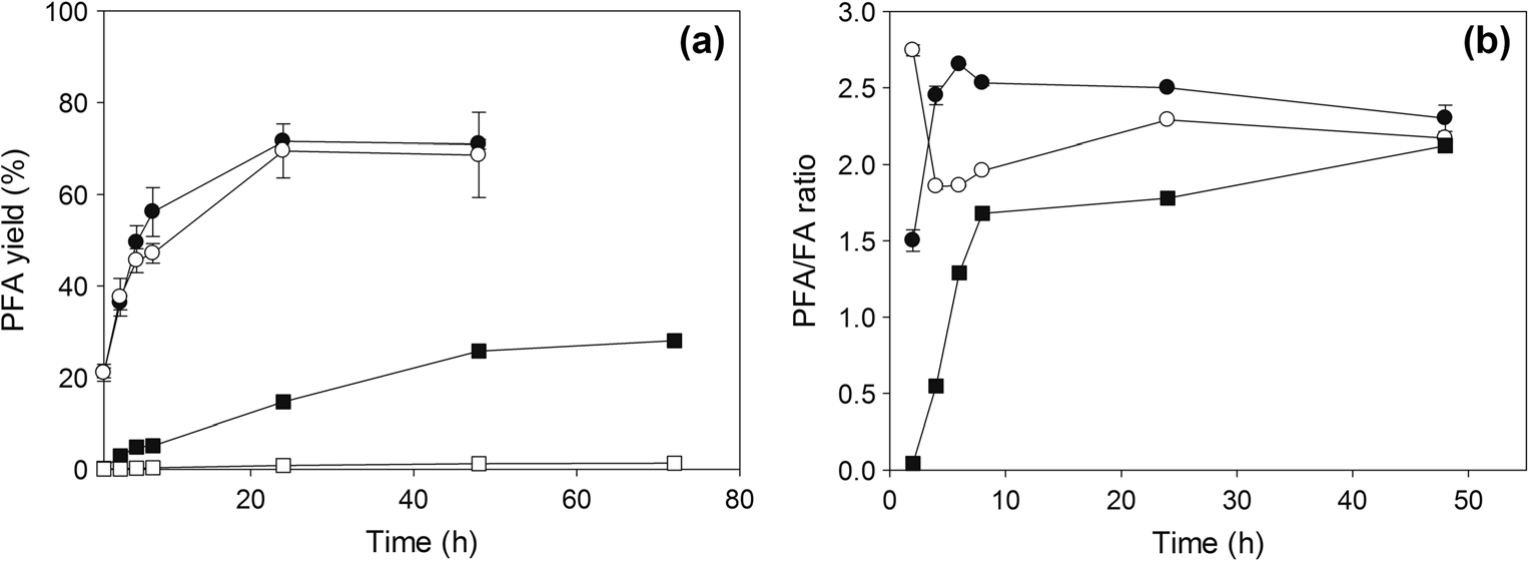
Effects of different donors and agitation on the **a** yield and **b** product selectivity. Reactions were performed by FaeB2 at optimal conditions. VFA (*black circle*), VFA and agitation (*white circle*) (1000 rpm), MFA (*black square*), and FA (*white square*)

The effect of different donors and agitation was studied for 96 h at optimal conditions using FaeB2 (Fig. 6). As expected, VFA was a more reactive donor, as the by-product vinyl alcohol tautomerizes to acetaldehyde, shifting the equilibrium towards transesterification. Agitation offered higher selectivity towards synthesis during the first 2 h of incubation probably due to the better contact of the biocatalyst with substrates and better mass and heat transfer. At these conditions, the PFA/FA ratio decreased with respect to time, while it was observed that it is stabilized at ~2.36 after 48 h independently of agitation. The same value is reached when MFA was used as donor, revealing that the synthetic specificity of FaeB2 is not affected significantly by the substitution on the feruloyl donor. Regarding the yield, VFA was fully converted to products fast (71.5% PFA after 24 h), while the reaction appeared much slower (25.8% PFA after 72 h) when MFA was used. Direct esterification of FA was negligible (1.4 after 96 h).

### Isolation of products and recovery of enzyme at optimal conditions

At the end of 24 h, the reaction mixture (500 μL) was separated into two phases. The upper organic phase contained only PFA, as VFA was converted completely (100%) by FaeB2, while the lower aqueous phase contained FA and the enzyme. The organic phase was evaporated, and PFA was recovered successfully (98.5%, 5.542 mg) and was subjected to identification by ^1^H NMR (chemicals shifts are reported in materials and methods). FA was collected after buffer exchange, while FaeB2 maintained its initial specific activity by 83%. In that context, detergentless microemulsions are proved to be an attractive system with environmentally friendly prospects as the high lipophilicity of PFA combined with the total conversion of VFA allows product separation with low energy costs. Moreover, the recovery and potential reusability of FaeB2 could reduce the cost of enzyme production.

### Antioxidant activity and cytotoxicity

**Fig. 7:**
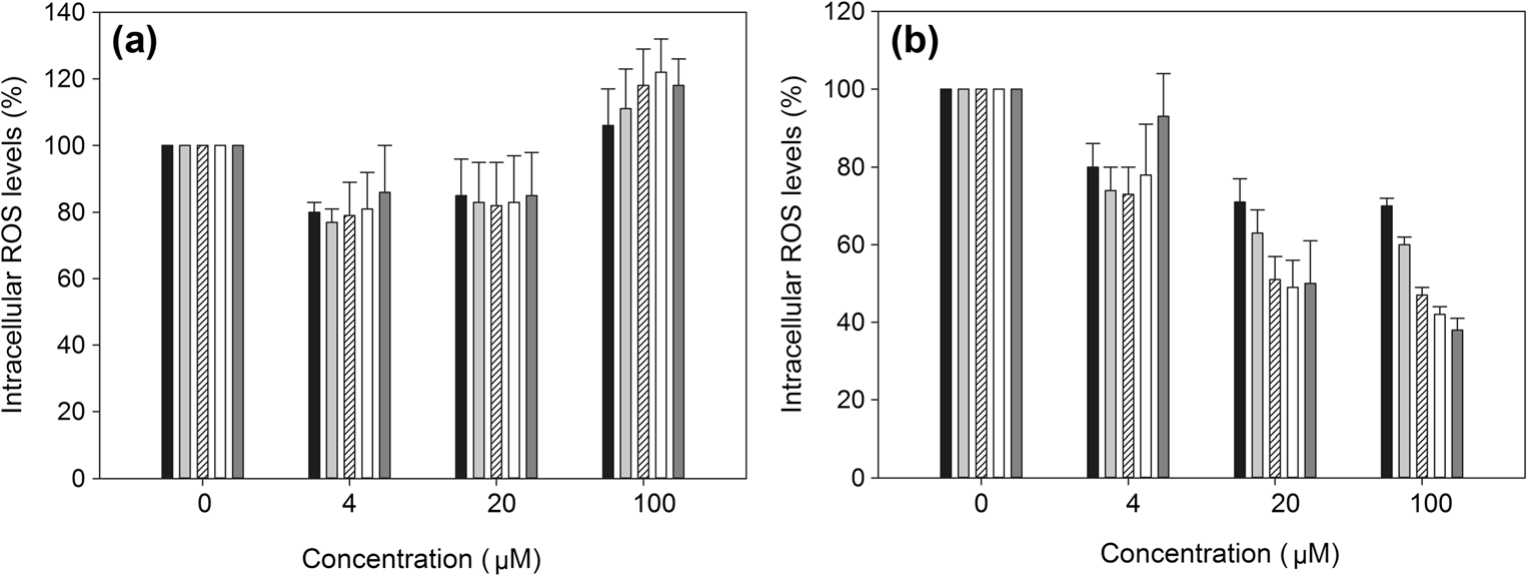
Antioxidant activity of **a** PFA and **b** FA determined with the DCFH-DA assay. *Black* 0.5 h, *light gray* 1 h, *striped* 3 h, *white* 6 h, and *dark gray* 24 h

The free radical scavenging activity of synthesized PFA as well as of its corresponding FA was investigated with the DPPH cell-free assay. Both compounds have significant scavenging activity, while FA was found slightly more potent. The half maximal inhibitory concentration (IC_50_), estimated after 3 h of incubation, was 329.9 μM for FA and 423.39 μM for PFA. Our observations are in accordance with previous studies reporting the scavenging ability of FA and its related esters (Kikuzaki et al. 2002). Following, the study of the antioxidant activity was aimed in a cell system based on HSFs. To this end, cytotoxic activity was determined by the MTT assay. Data indicate that both PFA and FA at concentrations ranging from 0.8 to 100 μM do not affect HSFs’ viability. At higher concentrations, PFA displayed a cytotoxic action (IC_50_ 220.23 μM), while FA was non-cytotoxic even at 500 μM, the highest concentration tested. These results are in agreement with previous reports on the effect of FA on the viability of human skin fibroblasts (Cos et al. 2002) and human embryonic kidney 293 (HEK293) cells (Bian et al. 2015). The DCFH-DA assay was employed at non-cytotoxic concentrations in HSFs. As shown in Fig. 7, FA reduced significantly the intracellular levels of ROS in a concentration-dependent manner, in accordance with previous data from a murine macrophage cell line (Nadal et al. 2016). PFA also provoked a reduction of intracellular ROS at concentrations ranging from 4 to 20 μM, while at 100 μM, no significant antioxidant effect was observed.

## Discussion

In this work, we demonstrated the potential of five FAEs derived from *M. thermophila* to synthesize the novel compound prenyl ferulate. Among them, FaeB2 proved to be the most efficient biocatalyst with attractive properties including high product selectivity (2.373), high yield (71.5% PFA), high overall yield (100%), low enzyme load (0.02 mg FAE/mL), reduced reaction time (24 h), and low operation temperature (30 °C). Results on the effect of solvent composition revealed that type A FAEs (FaeA1 and FaeA2) prefer an environment with higher water content (5.5%; system IV) than type B FAEs. Moreover, it was observed that in every optimization step, the product selectivity (PFA/FA ratio) was quite low for type A FAEs, without meaning that they are not active overall since the side hydrolysis was observed to be very robust for these enzymes. Subsequently, they showed low affinity towards lipophilic prenol comparing to type B FAEs and proved to be less efficient biocatalysts for the synthesis of PFA. Interestingly, each type B FAE demonstrated similar turnover rates (*k*_cat_) for both substrates, while each type A FAE had higher turnover rate for prenol comparing to VFA (Table 2). After optimization, the PFA/FA ratio was <1 only for FaeA1 and FaeA2 (0.782 and 0.239, respectively), while they appear to be more thermophilic enzymes with optimal temperature of 55 and 45 °C. On the contrary, type B FAEs are more mesophilic.

The synthetic activity and potential of tested FAEs are not directly correlated with their hydrolytic activity. All FAEs showed specific hydrolytic activities of the same magnitude (Table 1), while the transesterification potential of type B FAEs performed in detergentless microemulsions was generally twofold to threefold higher than the one of type A. This substantial difference could be attributed to structural differences. In most lipases, a mobile hydrophobic lid covers the substrate-binding site, and in this closed structure, the lipase is assumed to be inactive. Upon activation of the lipase by contact with a hydrophobic solvent or at a hydrophobic interface, the lid opens offering accessibility to substrate binding (Rehm et al. 2010). Although very few structures are available for FAEs, it is proved that the catalytic triad of the type A AnFaeA and the active site cavity is confined by a lid and a loop that confers plasticity to the substrate-binding site in analogy with lipases (Hermoso et al. 2004). What is surprising is the fact that FAE’s lid exhibited a high ratio of hydrophilic residues, keeping it in an open conformation that gives the typical preference of catalyzing the hydrolysis of hydrophilic substrates compared to lipases. According to this, the low product selectivity (PFA/FA ratio) of type A FAEs and the preference for environments with higher water content could be attributed to the presence of a higher percentage of hydrophilic amino acids in their lid comparing to the type B FAEs.

Glycosylation can affect the transesterification along with the enzyme properties. Among tested enzymes, FaeB2 and MtFae1a share the same primary sequence, while the latter has undergone glycosylation resulting to an observed MW of 39 kDa, instead of 33 kDa. The difference of the post-translational modification in their respective production hosts results in two biocatalysts with common origin but different performance. Specifically, both FAEs showed preference in the same media composition and water content (3.2%), while MtFae1a had 2.6-fold lower affinity towards VFA, is required at a 10-fold higher concentration in the reaction mixture, and operates optimally at alkaline pH. Interestingly, its affinity towards prenol is slightly higher (1.22-fold), and it appears more stable at very high acceptor concentrations (1.5–3 M). Finally, although both enzymes perform optimally at 30 °C, FaeB2 appears to inactivate at 35 °C, while MtFae1a has the same profile as at 30 °C. Overall, glycosylation offered increased stability regarding prenol, pH, and temperature but influenced negatively the process in terms of efficiency and cost, as the transesterification was characterized by lower substrate affinity for VFA, lower biocatalytic efficiency (260–560-fold decrease), limited yields (1.7-fold), and product selectivity (1.4-fold decrease).

In transesterification reactions catalyzed by FAEs, only the use of methyl activated donors is reported (Antonopoulou et al. 2016). In this work, for the first time, a vinyl donor was used in a FAE-catalyzed reaction. The use of vinyl activated donors is a common practice as they are more reactive offering high rates and reduced reaction times. Moreover, under normal conditions, the by-product vinyl alcohol tautomerizes to acetaldehyde and is easier to be removed comparing to methanol when MFA is used as donor. FaeB2 was the only enzyme that fully converted VFA to products after 24 h of incubation, while other enzymes such as FaeA1 reached very high overall yield (93.9%) at given time. As VFA and the transesterification product PFA have high and similar lipophilicity, a total conversion of donor relieves the process from laborious purification steps. On the other hand, it is evident that when MFA is used as donor, only 25.8% are converted to PFA after 72 h, indicating that separation, recovery, and reuse of substrate are essential additionally to increased operational costs (Fig. 6). There are numerous reports on the use of vinyl donors in lipase-catalyzed reactions (Chigorimbo-Murefu et al. 2009; Yang et al. 2010; Yu et al. 2010; Schär and Nyström 2016). Recently, a cutinase from *F. oxysporum* catalyzed the acylation of tyrosol using various vinyl esters with good yields (up to 60.7%) (Nikolaivits et al. 2016). However, the cost of the synthesis of an activated donor such as VFA is an issue to be addressed.

Using VFA as donor and at optimal conditions, the obtained yield is comparable with other reports on the synthesis of lipophilic feruloyl or other hydroxycinnamic acid derivatives catalyzed by FAEs. Very high yield (95% after 12 h) has been reported regarding the synthesis of diglycerol ferulates catalyzed by a FAE from *A. niger* (purified from the commercial preparation Pectinase PL “Amano”) (Kikugawa et al. 2012). The esterification of FA with glycerol yielded 81%, while esterification of *p*-coumaric acid yielded approximately 60% after 72 h using the same enzyme (Tsuchiyama et al. 2006, 2007). The transesterification of methyl sinapate with 1-butanol using AnFaeA resulted to 78% conversion after 120 h (Vafiadi et al. 2008b). All aforementioned modifications though were employed in systems where the acceptor is used in very high concentrations as solvent component. Although high concentrations of prenol lead to increased product selectivity, a limiting factor in the reaction is the inactivation of FaeB2 and other enzymes at concentrations higher than 1 M. Given this, a 71.5% yield of PFA after optimization is quite promising.

Detergentless microemulsions are a reaction system used in the vast majority of synthetic reactions based on FAEs as they do not affect enzyme activity (Topakas et al. 2003, 2004, 2005; Vafiadi et al. 2005, 2006a, b, 2008a, b; Couto et al. 2010, 2011). They form in ternary systems consisting of a hydrocarbon, a polar alcohol, and water representing thermodynamically stable and optically transparent dispersions of aqueous microdroplets in the hydrocarbon solvent. The droplets are stabilized by alcohol molecules adsorbed at their surface and possess spherical symmetry (Khmelnitsky et al. 1988). In the present reaction, we propose that the enzyme is enclosed and protected in the microdroplet, while the lipophilic donor VFA is present in the organic phase (comprising of *n*-hexane, *t*-butanol, and prenol). Microdroplets are stabilized by *t*-butanol, while the acceptor prenol (logP equal to 0.91) is mostly present in the organic phase as it is less polar than *t*-butanol (log P equal to 0.584). Given that, the target reaction takes places in the interface of the microdroplet when the enzyme gets in contact with VFA and prenol leading to transesterification. The side reaction of hydrolysis occurs when the enzyme gets in contact with VFA and water molecules. We propose that the transesterification product (PFA) is transferred in the organic phase immediately after its production due it its increased lipophilicity, which subsequently protects it from further hydrolysis. This is the main reason why this reaction keeps moving towards synthesis until equilibrium is reached.

An ideal solvent should offer attractive characteristics to the process such as low toxicity, easy product recovery, aiding substrate solubility, aiding the targeted reaction, and not affecting enzyme activity (Wei et al. 2003). The transesterification of VFA with prenol in detergentless microemulsions offers easy product separation and recovery, recycling of solvents, enzyme recovery, and reuse. Shifting the physicochemical equilibrium of the microemulsion and causing the formation of two separate phases, the lipophilic product PFA is encountered in the upper organic phase, while the hydrophilic FA and enzyme are found in the lower aqueous phase. Enzyme can be recovered and reused by ultrafiltration, while PFA and FA can be recovered by vacuum evaporation from their respective phase. A drawback of the composition of microemulsions is the lower solubility of VFA at room temperature, while they appear to enable a robust hydrolytic side reaction additionally to the target reaction. Nevertheless, the presence of water is essential for FAEs as they tend to be inactivate in pure hydrophobic media contrary to lipases. In enzymatic (trans)esterification, an amount of donor inevitably will get hydrolyzed due to the presence of water constituting product separation and purification essential.

A methodological dilemma in transesterification is whether as optimum parameter should be considered the one that offers higher concentration of desired product (or yield or rate) or the one that offers highest product selectivity. In the present optimization study, conditions that offered highest concentration of PFA were chosen as optimal, as parameter values that offered highest product selectivity (PFA/FA ratio) in most cases were detrimental to the yield. For instance, it was observed that lower water content (2%) offered higher PFA/FA ratio but decreased the rate and yield up to 50% for all FAEs except for FaeB1. Donor concentration did not affect product selectivity, probably because the presence of more VFA molecules close to the interface of the microemulsions has the same chance for transesterification and hydrolysis. Prenol concentration and enzyme load were factors that affected product selectivity the most. When prenol concentration is increased, more molecules are available near the interface of the organic and water phase of the microemulsion, allowing more frequent transesterification instead of hydrolysis. However, very high prenol concentration (>1 M) seemed to cause enzyme inactivation. In the case of enzyme concentration, optimal values for yield and product selectivity coincided. Finally, pH values offering high PFA/FA ratio resulted in lower yields for FaeA2 and FaeB1, while temperature, time, and agitation did not affect significantly the selectivity. Although FA production could be further minimized in an optimization study focused on highest selectivity, still some hydrolysis would be observed due to the inevitable presence of water sustaining the need of a purification step. In the same time, higher enzyme loads would be needed increasing the cost. Using FaeB2 at optimal conditions, hydrolysis was reduced 4.7-fold compared to the initial conditions. Moreover, detergentless microemulsions offer easy separation between PFA and FA, allowing the utilization of the latter for its beneficial effects as food additive, dietary supplement, etc.

Overall, this novel reaction can prove attractive for industrial utilization, as it offers an environmentally friendly substitute for existing synthetic reactions. The novel compound prenyl ferulate is a potential alternative to FA, showing high lipophilicity, similar antioxidant activity, and non-toxic effects at low concentrations.
